# Why are we addressing gender issues in vision loss?

**Published:** 2009-06

**Authors:** Paul Courtright, Susan Lewallen

**Affiliations:** Kilimanjaro Centre for Community Ophthalmology, PO Box 2254, Moshi, Tanzania.

**Figure:**
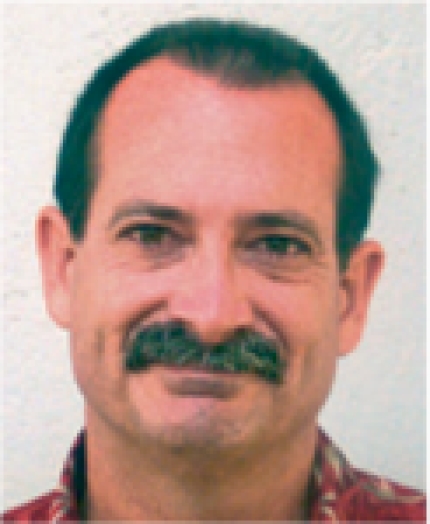


**Figure:**
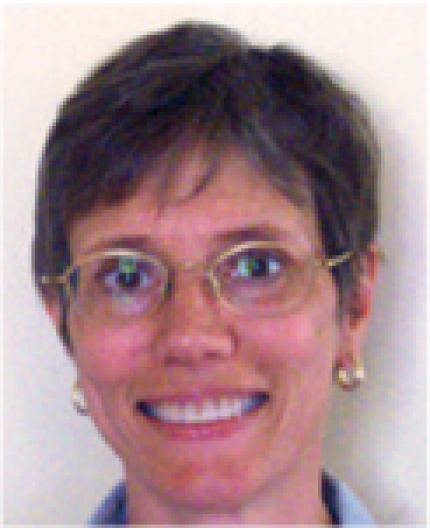


In the last decade, there has been increasing evidence that women are affected by blindness and visual impairment to a much greater degree than men. A systematic review of global population-based blindness surveys carried out between 1980 and 2000 showed that blindness is about 40 per cent more common in women compared to men (in persons older than 50 years).^1^ Since then, there have also been a number of large national surveys (for example, in Pakistan and Nigeria), as well as many rapid assessment of avoidable blindness studies (RAABs), which have confirmed the earlier findings. We now know that being a woman is a significant risk factor for some eye diseases; it is also an important factor in the use of eye care services:

- Women account for about 64 per cent of the total number of blind persons globally (a summary value).
- In many areas, men are twice as likely as women to be able to access eye care.^2^

**Figure 1:**
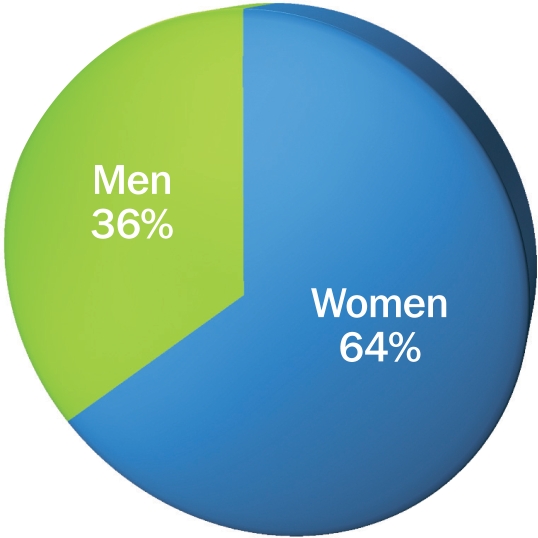
Estimated global blindness by gender (2001)^1^

The higher rates of blindness and visual impairment among women can be explored from three different perspectives: risk factors, access to services, and life expectancy.

**Figure:**
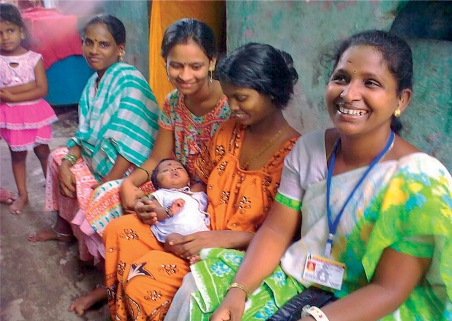
A female eye care worker (right) supports mothers in the Mumbai community where she lives. INDIA

Shilpa Vinod Bhatte

**Figure:**
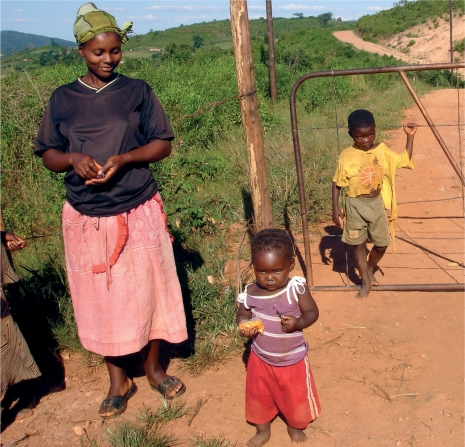
Women's responsibilities as child care providers often make it difficult for them to leave home and travel to where eye care is available. SWAZILAND

Daisy Wilson

1 **Risk factors.** Social and cultural differences between men and women can expose women to a greater risk of eye disease. For example, in countries where trachoma is endemic, the role of women as child care providers means they may be more likely to develop trachomatous trichiasis; this is due to their increased exposure to children with trachoma. Biological differences between men and women can also lead to increased risk of some eye diseases in women. For example, there is good evidence that women have a slightly higher incidence of cataract than men. Reasons for the higher incidence are not fully understood, but hormonal differences may contribute.^3^
2 **Access to services.** The social, cultural, and economic differences which exist between men and women also lead to reduced access to services for women. For example, women may not have freedom of movement, their need for eye care may not be considered as urgent or important as that of male family members, or they may not have financial decision-making authority within the family to pay for eye care services. In addition, women's child care responsibilities may make it difficult for them to leave home. Women's fears of how their vision loss may affect their status in the family may also make them downplay their loss of vision (and hence their need for eye care or surgery); some may prioritise their family's needs above their own need for eye care.
3 **Life expectancy.** It is important to remember that, in most cultures, women have a longer life expectancy than men. Since vision-related disorders increase with age, this will mean that there are more women with certain eye conditions, particularly those which occur late in life.

In recognition of these inequalities between the eye health of men and women, the theme of World Sight Day 2009 is ‘Gender and Eye Health’. The use of the word ‘gender’ in the theme is very specific: whereas the term ‘sex’ refers to the biological differences that make us male or female, ‘gender’ refers to the different roles women and men are expected to fulfil in their culture or society. As we saw above, these different roles often have a direct (and negative) impact on the eye health of women.

The following areas require attention if we are to address gender inequalities in eye health.

## Cataract

There is evidence that adult men and women still have unequal access to cataract services. Because women have a slightly higher incidence of cataract and tend to have longer life expectancy than men, women should account for 60–65 per cent of all cataract operations. However, recent analysis of 22 population-based studies in 17 low- and middle-income countries showed that, in all studies except one, more men than women received cataract surgery.^2^

There can be gender differences in the quality of cataract surgery people receive. In some settings, women are less likely to have intraocular lens (IOL) surgery compared to men. Since few settings routinely report outcome data, there is no information on the extent of this inequality.

At least three approaches have been shown to improve access to cataract surgery for women: bringing women and services together, counselling family members, and using women to reach women.

1 **Bringing women and services together.** In much of Africa and Asia, providing transport to hospital is essential for reaching older women in rural areas. Due to financial, social, and cultural constraints, these women are often unable to travel to a hospital to seek services. Studies of patterns of surgery utilisation, such as one carried out in Tanzania,^4^ also confirm the importance of outreach activities to reach women with cataract.
2 **Counselling family members.** Older women in most low- and middle-income countries do not have the ability to make individual decisions regarding their own health. In most cases, decision making is the remit of husbands or sons. An older woman will not be in a position to accept surgery unless her family supports her to do so. Counselling of family members, even when surgery is provided at a highly subsidised price or free of charge, appears to be an important approach where women are concerned.^5^
3 **Using women to reach women.** As colleagues from Pakistan, Egypt, and India have noted in this issue, the potential contribution of woman to woman contact can be considerable as it tends to build trust between women and health care providers.

For all eye care providers, a critical first step is to start monitoring the use of services and outcome of surgery separately for men and women. A critical review of routine programme data can be an extremely powerful tool for finding the magnitude of the problem and for encouraging programme staff to start exploring solutions.

## Trachoma

Trachoma is one of the few ‘life cycle’ eye diseases affecting people of all ages in endemic areas of the world. Recent work in Southern Sudan has demonstrated trichiasis in children, with girls being 1.5 times more likely to have trichiasis than boys.^6^ While there may be some biological reason that girls develop a more intense response to *Chlamydia trachomatis*, the major reason that females (of all ages) are 1.8 times more likely to have trichiasis compared to men^7^ relates to their gender roles and responsibilities as child care providers (see page 22).

As the SAFE strategy indicates, addressing trachoma requires planners and health care providers to consider all of the various ways we reach into communities and address the needs of women and girls. Interventions can be related to water use, latrines, distribution of antibiotics, or surgery for trichiasis. The recently published manual *Women and Trachoma: Achieving Gender Equity in Trachoma Control* (see page 32) aims to provide those involved in trachoma control with strategies to ensure that the current inequality in the burden of trachoma becomes history.

## Childhood blindness

Nutritional blindness (such as vitamin A deficiency) was once the most common cause of blindness in children; it has now become rare^8^, except in areas of extreme poverty or recent unrest. As a result, congenital and developmental cataract has now emerged as a common cause of blindness in various settings in the world. With the establishment of paediatric ophthalmology tertiary centres, reports on utilisation of services, not normally available before, have now been generated. These reports generally show significantly more boys than girls receiving surgery for congenital or developmental cataract. As Bronsard and Shirima note in their article in this issue, although gender inequality in use of services (surgery or postoperative spectacles and low vision devices) may exist, it need not be inevitable. Recognising gender inequalities in service uptake is the first step to finding ways to correct it.

## Glaucoma and diabetic retinopathy

Although the gender issues surrounding glaucoma and diabetic retinopathy are not explored in this issue, this does not mean they should be ignored.

There is clear evidence from eastern Asia that women have a higher incidence of primary angle-closure glaucoma (PACG) compared to men.^9^ There have not been adequate investigations to determine whether women with PACG have equal access to services (or similar outcomes) as men.

There appears to be no sex-specific difference in incidence of primary open-angle glaucoma, but the few studies of service use suggest that men are more likely than women to receive surgical services.^10^

Hospitals providing medical or surgical treatment for glaucoma need to start monitoring use of these services by sex.

Diabetes is a growing epidemic throughout much of Asia and Africa, but there is inadequate data to suggest differences between men and women in incidence or utilisation of services for diabetic retinopathy.

Glaucoma and diabetes are growing priorities that could be managed more equitably and effectively if we understood how people used the eye care services we offer.

## The way forward

All of the articles in this issue should stimulate us to take some steps, the first of which is to understand these problems at community, country, and global level. It is at the community level where we will make the biggest impact; we should remember that our service delivery systems need to consider the needs of women as well as men. At the country and global level, as recently recommended by WHO, we should get into the habit of providing reports that are disaggregated by sex. This is not necessarily onerous. In fact, most health care systems already routinely record sex; we just need to make sure that this information is not lost along the way in our reporting.

The World Sight Day 2009 theme of ‘Gender and Eye Health’ is both a challenge and an opportunity for the prevention of blindness community. Our health care environments are undergoing change: patient expectations are changing, technology is changing, and communities are changing. Ensuring that our eye care programmes address gender inequalities will enable all people – men as well as women, boys as well as girls – to receive the best we have to offer.

**What can we do?**

- When **consulting with a community** about eye care programmes or services, include women and encourage the community to involve women in decision-making.
- When **designing services and delivering them**, incorporate assistance to women, for example by providing transport to clinics or using female health workers where cultural or religious taboos exist.
- When **conducting research or monitoring programmes**, disaggregate the data by sex.
